# Seasonal variation in abundance and blood meal sources of primary and secondary malaria vectors within Kilombero Valley, Southern Tanzania

**DOI:** 10.1186/s13071-022-05586-z

**Published:** 2022-12-20

**Authors:** Godfrey C. Katusi, Marie R. G. Hermy, Samwely M. Makayula, Rickard Ignell, Nicodem J. Govella, Sharon R. Hill, Ladslaus L. Mnyone

**Affiliations:** 1 Department of Environmental Health and Ecological Sciences, Ifakara Health Institute, Off Mlabani Passage, Ifakara, P.O. Box 53, Morogoro, Tanzania; 2 Department of Microbiology, Parasitology and Biotechnology, College of Veterinary Medicine and Biomedical Sciences, Sokoine University of Agriculture, P.O. Box 3019, Morogoro, Tanzania; 3 Disease Vector Group, Unit of Chemical Ecology, Department of Plant Protection Biology, Swedish University of Agricultural Sciences, P.O. Box 190, 234 22 Lomma, Sweden; 4 Pest Management Centre, Sokoine University of Agriculture, P.O. Box 3110, Morogoro, Tanzania; 5 School of Life Sciences and Bioengineering, Nelson Mandela African Institution of Science and Technology, Arusha, Tanzania

**Keywords:** Mosquito, Abundance, Blood meal source, Host preference, Sporozoites

## Abstract

**Background:**

Existing control tools have significantly reduced malaria over the past two decades. However, progress has been stalled due to increased resistance in primary vectors and the increasing role of secondary vectors. This study aimed to investigate the impact of seasonal change on primary and secondary vector abundance and host preference. Understanding the impact of seasonal dynamics of primary and secondary vectors on disease transmission will inform effective strategies for vector management and control.

**Methods:**

Vector abundance was measured through longitudinal collection of mosquitoes, conducted monthly during the wet and dry seasons, in Sagamaganga, a village in the Kilombero Valley, Tanzania. Mosquitoes were collected indoors using CDC light traps and backpack aspirators, and outdoors using resting buckets baited with cattle urine. In addition, a direct measure of host preference was taken monthly using human- and cattle-baited mosquito electrocuting traps. A host census was conducted to provide an indirect measure of host preference together with monthly blood meal source analysis. All collected mosquitoes were assayed for *Plasmodium* sporozoites.

**Results:**

A total of 2828 anophelines were collected, of which 78.5% and 21.4%, were primary and secondary vectors, respectively. The abundance of the primary vectors, *Anopheles arabiensis* and *Anopheles funestus,* and of the secondary vectors varied seasonally. Indirect measures of host preference indicated that all vectors varied blood meal choice seasonally, with the direct measure confirming this for *An. arabiensis*. All anopheline mosquitoes tested negative for sporozoites.

**Conclusions:**

At the study location, the abundance of both primary and secondary vectors changed seasonally. Indirect and direct measures of host preference demonstrated that *An. arabiensis* varied from being zoophilic to being more opportunistic during the wet and dry seasons. A similar trend was observed for the other vectors.

**Graphical Abstract:**

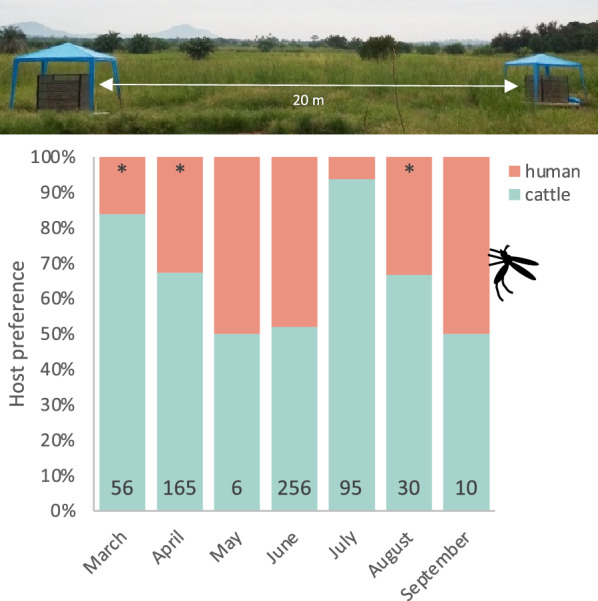

## Background

The general burden of malaria in sub-Saharan Africa (SSA) has decreased significantly over the last decade [1, 2]. This reduction is largely due to improved coverage of long-lasting insecticidal nets (LLINs) and indoor residual spraying (IRS), as well as early diagnosis and treatment of malaria with artemisinin-based combination therapy [1, 3–6]. This commendable reduction has, however, recently stalled [2], predominantly due to increasing behavioral and physiological resistance in primary vectors [7–9] and the increasing role of secondary vectors in disease transmission. The existing frontline vector control tools are not effective at targeting the resistant primary and the emerging secondary vectors [10, 11].

Historically, primary malaria vectors have been responsible for approximately 95% of the transmission in SSA [12, 13]. Over the past two decades, however, there has been a considerable change in the composition and designation of primary malaria vector species throughout this region [14–16], with secondary vectors now known to contribute to malaria transmission, particularly due to their exophilic and exophagic behaviors [17–19]. Moreover, recent studies, though few in number, indicate that secondary vectors are increasingly reported as contributing to ongoing residual malaria transmission [18, 19]. Current understanding of the ecology of both primary and secondary vectors, and how these respond to seasonal variation, remains limited but is critical for the design and allocation of interventions. Thus, updated regional information on the seasonal activity of, and malaria transmission intensity in, primary and secondary vector populations is necessary to help strengthen knowledge on how to tackle these vectors [20–22].

In Tanzania, and other parts of SSA, *Anopheles arabiensis* and *Anopheles funestus *sensu stricto (*An. funestus *s.s.) are the primary malaria vectors [23–25]. These vectors differ in their feeding preference and malaria transmission efficiency, with *An. funestus* s.s. being more anthropophilic than *An. arabiensis*, which rather are more opportunistic [11, 26, 27]. Due to this difference in host preference, *An. funestus *s.s. is considered to be the predominant malaria vector in the area [24, 28]. Control of these and other vectors is limited, due to increased insecticide and behavioral resistance [8] and a current dearth of knowledge concerning their ecology and population dynamics at a local scale, both of which are pertinent for improving surveillance and control strategies to achieve malaria elimination. In this study, a longitudinal investigation was performed to determine (i) the seasonal abundance and blood meal sources, as a proxy for host preference, of primary and secondary malaria vector species in Sagamaganga village, Kilombero Valley, Tanzania; and (ii) the potential contribution of secondary malaria vectors in transmitting malaria parasites. We discuss our results in the context of vector ecology and its contribution to future sustainable vector control strategies and management.

## Methods

### Study area

**Fig. 1:**
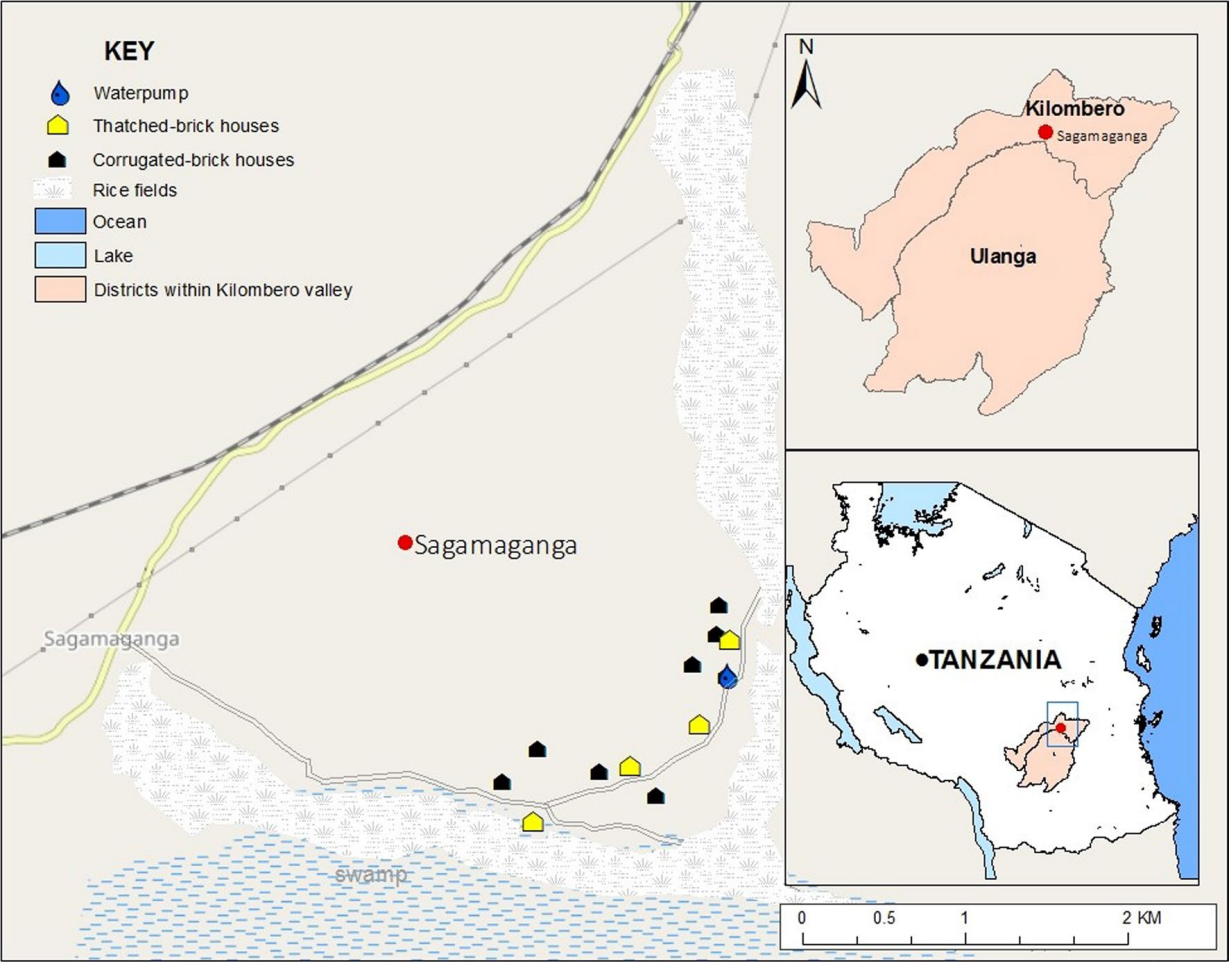
Map showing the study village, Sagamaganga, in south-eastern Tanzania

This study was conducted in Sagamaganga village (8°3′50.352″ S, 36°47′46.254″ E), in the Kilombero Valley, south-eastern Tanzania (Fig. 1). The valley lies 300 m a.s.l. The average annual temperature ranges between 20 °C and 32 °C, with the annual rainfall ranging between 1200 and 1800 mm [29]. The general wet and dry seasons are from February to June and July to January, respectively. The main economic activities are agricultural, and include rice cultivation and livestock keeping. The most common domestic animals include cattle, goats, sheep, chickens and dogs. The malaria prevalence in Kilombero valley has generally decreased over the past decade, notably from 14% in 2011 to 11% in 2017 [30, 31].

### Selection and characteristics of study households

The global positioning system (GPS) coordinates of all households, in the part of Sagamaganga village where the study was conducted, were recorded using a hand-held GPS (Garmin GPSMAP 60CSx; Garmin International Inc., Olathe, USA) before the final selection of study households. Ten study households were selected from the list of 33 houses in the part of the village with high livestock keeping, using a simple random sampling technique by R statistical software (version 3.6.2; [47]). All study households had mud-brick walls and open eaves. Four households had thatched roofs, while the remaining six had corrugated iron roofs. One household had no cattle shed, but was surrounded by cattle sheds from neighboring households. The number of occupants per household varied from one to five. Depending on the number of beds, each household was provided with one to three new LLINs (Olyset; A to Z Textiles Mills, Arusha, Tanzania). In half of the study households, chickens were kept in the living rooms during the night. In all study households, cooking was done outside.

### Host census

The host census data were obtained before the onset of the study by surveying the household owners within the study area regarding the number of household occupants and the number of animals owned and present during the study.

### Adult mosquito collections

**Fig. 2:**
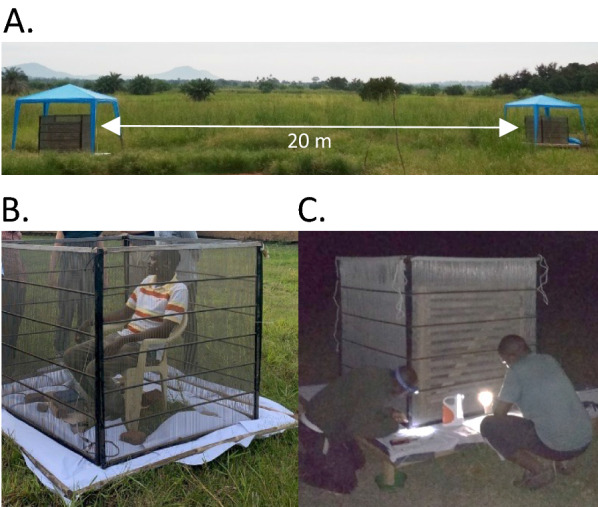
The overall set-up (**a**), a person in the trap during the day for clarity (**b**), and a night-time photo showing the actual collection of electrocuted mosquitoes around the MET trap baited with a calf (**c**). MET, Mosquito electrocuting trap

Mosquitoes were collected from inside and outside the study households. Indoor collections were conducted using one US Centers for Disease Control and Prevention (CDC) light trap (model 512; John W. Hock Company, Gainesville, FL, USA) per household [32], and a CDC backpack aspirator (John W. Hock Company) [33]. Outdoor collections were conducted using resting buckets (black plastic, 25.4 × 43 cm, diameter × height) baited with 24-h-aged cattle urine [34, 35], and two mosquito electrocuting traps (METs) baited with either a male human volunteer (METh) or a calf (METc) [36]. All collections were done in 2019, over a 6-month period across the wet and dry seasons, on 6 consecutive nights per month. Indoor collections using CDC light traps and backpack aspirator as well as outdoor collections using resting buckets were conducted during the first 5 nights per month, with MET trapping done on the sixth night of each month to assess host preference. The CDC light traps were suspended approximately 1.2 m above the ground next to a person sleeping under an LLIN and were turned on at 18:00 h and off at 06:00 h by a trained house occupant. The CDC backpack aspirator was used to collect mosquitoes resting on inner walls and ceilings of each house in the morning (06:00–09:00 h) for approximately 10 min. Resting buckets, lined with wet black cloth, one per household, were placed 5 m from the household and cattle shed(s), to collect outdoor resting mosquitoes. Each bucket was baited with 250 ml of 24-h-aged cattle urine collected in plastic cups. Mosquitoes inside the resting buckets were collected using a CDC backpack aspirator between 06:00 h and 09:00 h. The METs, baited with a human volunteer or a calf, were placed 20 m apart and approximately 100 m upwind of the households (Fig. 2). Both the human and calf weighed approximately 70 kg, with the same individuals used throughout the study. The METs were placed on wooden platforms raised 20 cm above the ground. The stand legs of each platform were kept inside water-filled plastic basins to prevent ants from interfering with the collected mosquitoes. During each experimental night, trapping with METs was conducted for 12 h from 18:00 h to 06:00 h. The traps were turned off every 45 min to allow 15 min for the collection of electrocuted mosquitoes.

### Mosquito species, blood meal and sporozoite analyses

Collected mosquitoes were killed with chloroform, identified using morphological identification keys [37, 38] and sorted based on their sex and abdominal status (fed and unfed). Mosquito species identified as *Anopheles gambiae *sensu lato (*An. gambiae* s.l.; *n* = 1713) and *An funestus* group (*n* = 507) were individually preserved in 1.5-ml Eppendorf tubes containing silica gel and submitted to the laboratory for further species-specific identification by multiplexed PCR [39–42].

Blood meal content in mosquitoes was analyzed by enzyme-linked immunosorbent assay (ELISA; Carramore International Ltd, Holmfirth, UK) using the abdomens of all blood-fed primary and secondary malaria vectors (*n* = 562) [43, 44]. Antisera from humans, cattle, goats, chickens, dogs (immunoglobulin G [IgG] identifiers; KPL, Gaithersburg, MD, USA) and sheep (VWR, Stockholm, Sweden) were used. Circumsporozoite ELISA (IgG identifiers, KPL) of heads and thoraces of all primary and secondary malaria vectors (*n* = 2828) were also conducted [45]. To avoid false positives, the ELISA lysate was heated to 100 °C for 10 min, to ensure total elimination of heat-liable non-*Plasmodium falciparum* antigens [46].

### Statistical analysis

Monthly variations in mosquito collections with CDC light traps, resting buckets and one backpack aspirator were analyzed using generalized linear mixed models (GLMMs) with R statistical software version 3.6.2 [47]. Since the data were zero-inflated and over-dispersed, as confirmed by the Shapiro test, a GLMM was used with the Template Model Builder package *glmmTMB* and a negative binomial distribution, as well as extensions to accommodate the zero-inflation and over-dispersion of the data [47, 48]. Following an initial modeling including “Month,” “Household IDs,” “Trap type,” “Location of the trap,” “Roof type,” “Feeding status,” “Number of cattle sheds,” “Distance of cattle sheds from the house,” “Number of cattle,” “Number of goats,” “Number of sheep,” “Number of chickens,” “Presence of chickens inside the house,” “Use of bed nets,” “Number of occupants in the house,” “Insecticide applied to cattle” and “Repellents applied to children” as fixed effects and “Date” treated as a random effect, those effects without significance and no interaction with other effects were removed until a low and stable second-order Akaike information criterion (AICc) was achieved, and the residual deviance approached the degrees of freedom. Please note that as all houses included in this study had open eaves and mud brick walls, these characters were not included in the analyses. In the final model addressing seasonal change in abundance, “Month,” “Location of the trap,” “Trap type” and “Household IDs” were treated as fixed effects, while “Date” was treated as a random effect. Separate analyses were performed for primary and secondary malaria vectors. In the final model addressing host preference, which included only blood-fed females, host preference was estimated as the percentage of fully fed mosquitoes for a given blood meal. A GLMM (*lme4* package, [47]) was used with a negative binomial distribution and accounting for over-dispersion to analyze the variation in host preference of fed mosquitoes across seasons, as well as across the locations (indoor/outdoor). The fixed effects were “Month,” “Location of the trap,” “Trap type” and “Household IDs,” while “Date” was treated as a random effect. No statistical analysis was conducted for sporozoite assays, as all mosquitoes tested negative.

### Ethics approval and consent to participate

The ethics approval to conduct this study was obtained from the Institutional Review Board of the Ifakara Health Institute (Ref. IHI/IRB/EXT/No: 23 – 2019), and the Medical Research Coordination Committee at the National Institute for Medical Research (NIMR) (Ref. NIMR/HQ/R.8a/Vol.IX/3085). Before data collection, community meetings were conducted to explain the purpose and data collection procedures. Signed informed consent was obtained from the heads of all study households. All study households were provided with a sufficient number of LLINs (Olyset® nets).

## Results

### Overall mosquito abundance

**Fig. 3:**
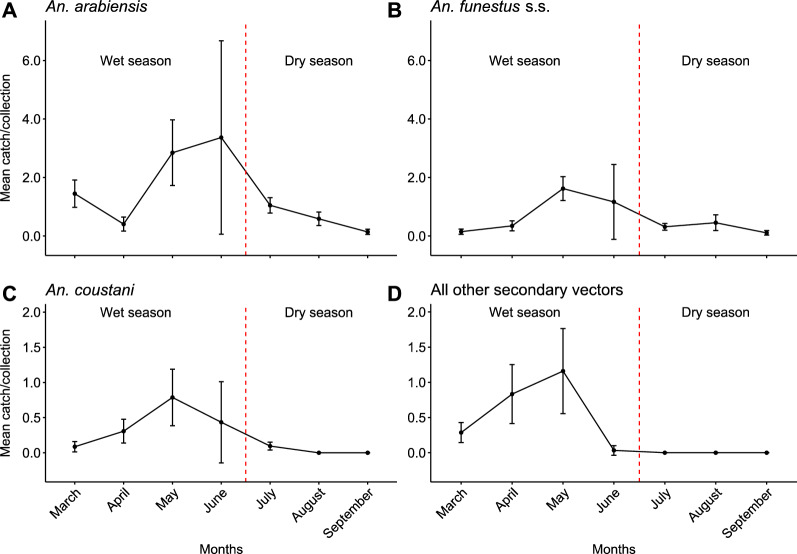
Seasonal change in the abundance of primary and secondary malaria vectors during the study period.** a**,** b** The primary vectors, **c** the main secondary vector, **d** other secondary* Anopheles* vector species (e.g. *An. squamosus*, *An. pharoensis*, *An. ziemanni*, *An. rufipes*, *An. wellcomei*, *An. tenebrosus*)

Combined across all the trapping collections, a total of 19,586 mosquitoes were caught and identified, of which 5877 (2828 females, 3049 males) were anophelines and 13,709 (9967 females, 3742 males) were culicines. The 2828 female primary and secondary vectors assayed by PCR were identified to be *An. arabiensis*, *An. funestus *s.s., *An. leesoni*, and *An. rivulorum* (Table 1)*.* The primary vectors, *An. arabiensis* and *An. funestus* s.s., collected with CDC light traps, backpack aspirators, and outdoor resting boxes were more abundant than the secondary vectors (*P* = 0.020). Of the secondary vectors, the dominant species were *An. coustani* and *An. squamosus*. The overall abundance of the anophelines (Table 1) varied across seasons (*χ*_1_^2^ = 104.06, *P* < 0.001), with significantly higher numbers collected during the wet season compared to the dry season, irrespective of the trap type (GLMM: OR = 5.10, *P* < 0.001, Fig. 3). During the wet season, the abundance of vectors outdoors increased by twofold relative to indoors (GLMM: OR = 2.42, *P* < 0.001). Roof type was found to have no effect of mosquito abundance in the GLMM.

**Table 1:** Malaria vectors collected indoors and outdoors during the wet and dry seasons

	Season (months)	Location	Collection/ trapping method	Primary* Anopheles* vector species		Secondary* Anopheles* vector species									
				*An. arabiensis*	*An. funestus* sensu stricto	*An. leesoni*	*An. rivolurum*	Unknown *An. funestus*	*An. coustani*	*An. squamosus*	*An. pharoensis*	*An. ziemanni*	*An. rufipes*	*An. tenebrosus*	*An. welcomei*
Household collections	Wet (March-June)	Indoor	CDC	658 (332^a^)	123 (109^b^)	84	36	23	155 (438)	153 (22)	105 (700)	13 (34)	9 (0)	6 (1)	2 (0)
		Indoor	BP	30 (21ª)	13 (2^b^)	16	2	8	15 (78)	13(0)	10 (16)	-	0 (1)	–	–
		Outdoor	RB	118 (98ª)	9 (23^b^)	21	4	12	20 (23)	4 (0)	24 (87)	-	0 (3)	4 (3)	–
	Dry (July-September)	Indoor	CDC	211 (209ª)	52 (58^b^)	34	16	5	19 (12)	–	–	–	–	–	–
		Indoor	BP	40 (16ª)	0 (2^b^)	6	1	1	–	–	–	–	–	–	–
		Outdoor	RB	39 (21ª)	1 (1^b^)	3	2	0	1	–	–	–	–	–	–
Host preference collection	Wet (March-June)	Outdoor	METc	293 (283ª)	0 (18^b^)	1	5	0	25 (71)	–	7 (43)	1 (0)	–	1 (0)	–
		Outdoor	METh	189 (176ª)	6 (8^b^)	1	1	0	7 (26)	–	0 (40)	–	–	2 (0)	–
	Dry (July-September)	Outdoor	METc	114 (14ª)	1 (13^b^)	5	5	1	5 (15)	–	4 (24)	–	–	–	–
		Outdoor	METh	21 (4ª)	5 (1^b^)	3	1	0	2 (1)	–	1 (2)	–	–	–	–
Total mosquitoes collected			2828	1713 (1174ª)	210 ( 235^b^)	174	73	50	249 (664)	170 (22)	151 (912)	14 (34)	9 (4)	13 (4)	2 (0)

Values in table are the number of mosquitoes collected, with the values in parentheses indicating the number of male mosquitoes

*CDC* CDC light traps, *BP* CDC backpack aspirator, *RB* Resting buckets, *METc *mosquito electrocuting trap with cattle as bait, *METh *MET with human as bait

^a^Males of the *An. gambiae* complex

^b^Males of the *An. funestus* complex

### Seasonal change in the abundance of primary and secondary malaria vectors

*Anopheles arabiensis* mosquitoes were collected in significantly higher numbers throughout the seasons (GLMM: odds ratio [OR] = 5.03, *P* = 0.015) compared to that of other vectors, peaking during May (*χ*_1_^2^ = 2.84, 95% confidence interval [CI]: 1.72–3.97) and June (*χ*_1_^2^ = 3.37, 95% CI: 0.05–7.67) (Fig. 3a). The abundance of *An. funestus* s.s. and *An. coustani* showed similar seasonal patterns as *An. arabiensis*, peaking in May (*χ*_1_^2^ = 1.62, 95% CI: 1.2–2.0; *χ*_1_^2^ = 0.78, 95% CI: 0.4–1.2) and June (*χ*_1_^2^ = 1.17, 95% CI: 0.1–2.5; *χ*_1_^2^ = 0.43, 95% CI: − 0.14 to 1.01) (Fig. 3b, c). In contrast, the abundance of the remaining secondary vectors declined significantly in June, to a level maintained throughout the dry season (Fig. 3d). The rate of increase in abundance of all secondary vectors appeared constant from March to May, whereas the abundance of the primary vectors significantly increased at a faster rate later in the season between April and May (GLMM: OR = 3.33, *P* < 0.001) (Fig. 3).

### The effect of host prevalence on mosquito abundance

The host census revealed an unequal abundance (Table 2) and distribution of potential human and animal hosts in the study village. Households with > 30 cattle had significantly higher numbers of primary (by approx. twofold) and secondary (by approx. fivefold) vectors compared to those with zero to 30 cattle (GLMM: OR = 1.98, *P* = 0.006 and OR = 4.64, *P* = 0.003, respectively). Moreover, households with 11–50 sheep had a significantly higher number of primary and secondary vectors compared to those without sheep (GLMM: OR = 2.81, *P* = 0.006 and OR = 4.77, *P* = 0.037, respectively). Approximately 61% (95% CI: 56.7-64.7%) of the human-fed primary and secondary vectors were collected in households with > 30 cattle, while the remaining vectors (95% CI: 35.6–43.6%) were collected in households with a lower number of cattle. Neither the presence nor the abundance of any of the other remaining hosts, including chickens, were found to affect mosquito abundance.

**Table 2:** The effect of host prevalence on primary and secondary malaria vector abundance

Household number	Host abundance (*n*)							Primary vector abundance						Secondary vector abundance					
	Humans	Bovine	Chickens	Sheep	Dogs	Cats	Goats	Total (*n*)	Mean	RR^a^	Lower^a^	Upper^a^	*P*-value^a^	Total (*n*)	Mean	RR^a^	Lower^a^	Upper^a^	*P*-value^a^
5	18	30	18	17	6	1	0	336	3.73	1.00	–	–	N/A	152	1.69	1.00	–	–	N/A
1	22	> 200	> 35	60	20	10	0	152	1.69	0.55	0.33	0.91	0.0195	54	0.60	0.40	0.16	0.96	0.0395
2	15	> 70	> 50	0	0	3	0	169	1.88	0.51	0.31	0.84	0.0078	40	0.44	0.13	0.05	0.35	0.0001
3	23	> 58	> 70	10	5	3	6	177	1.97	1.14	0.70	1.86	0.6045	64	0.71	0.34	0.14	0.84	0.0196
4	14	10	> 35	14	2	4	0	173	1.92	0.67	0.41	1.10	0.1127	33	0.37	0.15	0.06	0.41	0.0002
6	5	0	20	0	0	0	0	119	1.32	0.31	0.19	0.52	0.0000	49	0.54	0.29	0.12	0.72	0.0074
7	13	35	30	0	1	2	0	151	1.68	0.83	0.51	1.36	0.4561	74	0.82	0.60	0.25	1.45	0.2558
8	22	20	25	0	3	0	0	66	0.73	0.26	0.15	0.45	0.0000	22	0.24	0.11	0.04	0.32	0.0000
9	13	> 20	> 25	0	2	2	2	116	1.29	0.46	0.28	0.77	0.0031	33	0.37	0.16	0.06	0.42	0.0002
10	18	9	> 30	0	4	3	0	109	1.21	0.39	0.23	0.65	0.00	32	0.36	0.13	0.05	0.35	0.00

*GLMM* Generalized linear mixed model, *RR* relative risk

^a^Household number 5 was used as reference in the GLMM

### Blood meal source

As a proxy for host preference, all blood-fed *Anopheles* mosquitoes (*n* = 562) were analyzed for meal source (Table 3). Most blood-fed mosquitoes were caught in the resting buckets and the CDC light traps. The majority of the identified blood meals in *An. arabiensis* were bovine (42%) and sheep (52%), with only 2.8% of the mosquitoes having fed on a human (Table 3). In contrast, the majority of *An. funestus* s.l. fed on humans, while the rest fed on cattle or both human and cattle (Table 3). The blood meal analysis of the secondary vectors identified bovine as the primary blood meal source, with few blood meals from humans and other hosts (Table 3). Neither primary nor secondary vectors were found to contain chicken blood, despite this being the second most abundant vertebrate in the area (Table 3).

**Table 3:** Blood meal sources identified from primary and secondary malaria vectors

* Anopheles* vector species	Blood meal sources										
	Bovine	Human	Dog	Goat	Sheep	Bovine/Human	Bovine/Dog	Bovine/Goat	Human/Dog	Goat/ Dog	Unknown
*An. arabiensis*^a^	138	13	5	4	172	1	–	–	–	–	16
*An. funestus*^a^	5	13	–	–	–	1	–	–	–	–	–
*An. lessoni*	34	10	5	1	–	–	–	–	–	–	–
*An. rivolurum*	3	1	–	–	–	1	1	–	–	–	–
*Unknown An. funestus* sensu lato	13	1	1	1	–	–	–	–	–	–	–
*An. squamosus*	33	5	1	–	–	–	–	2	1	–	–
*An. coustani*	31	2	–	–	1	–	–	–	–	1	–
*An. pharoensis*	35	6	–	–	–	–	–	–	–	–	–
*An. ziemanni*	3	–	–	–	–	–	1	–	–	–	–
Total	295	51	12	6	173	3	2	2	1	1	16

Values in table are the number of blood-fed mosquitoes per* Anopheles *species and blood meal source

^a^Primary vectors

**Fig. 4:**
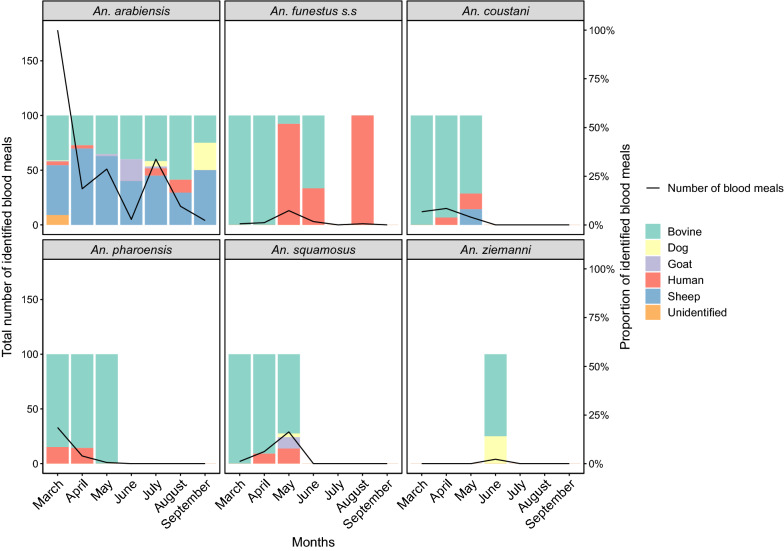
Seasonal change in the proportion of blood meals from various hosts. Blood meals were determined by enzyme-linked immunosorbent assay from bovine, dog, goat, and human hosts

Blood meal source varied significantly over the seasons (*χ*_1_^2^ = 18.81, *P* = 0.016), and the number of fed mosquitoes was approximately fivefold higher during the wet season than during the dry season (GLMM: *P* < 0.001) (Fig. 4). There was no association between location (indoor/outdoor) and blood meal source imbibed by fed mosquitoes (*χ*_1_^2^ = 4.86, *P* = 0.772). The balance between the blood meal sources in *An. arabiensis* changed across the seasons, with bovine blood meals dominating during the late wet and beginning of the dry seasons (May to August), while before and after this period sheep blood was predominant (Fig. 4). Although only a low number of blood-fed *An. funestus* s.s. were caught (Table 3), a large proportion of these were found to have human blood meals between May and August, while prior to this period the dominant blood source was bovine (Fig. 4). The majority of secondary vector species were only found to contain blood meals between March and May, except for *An. ziemanni*, which was only present in June (Fig. 4). While the majority of these blood meals were bovine, other sources, including human, were found (Fig. 4). The number of household occupants and insecticide treatment of the livestock (3–6 times per month, every 3 months) had no effect on mosquito blood meal source in the GLMM.

### Host preference analysis

**Fig. 5:**
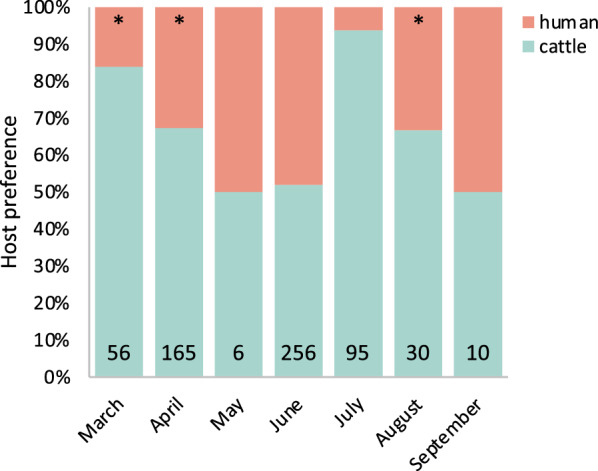
Seasonal change in the host preference of *Anopheles arabiensis* as assessed by mosquito electrocuting traps

Mosquito electrocuting traps, baited with either a human or a calf, were used to directly assess host preference. Of the 707 *Anopheles* mosquitoes collected using METs outdoors, the highest number of primary vectors were identified as *An. arabiensis* (94.6%, *n* = 617; 95% CI: 92.5–96.2%) with only a few *An. funestus* s.l. (5.3%, *n* = 35; 95% CI: 3.8–7.4%). Among the collected secondary vectors, *An. coustani* was the most abundant (70.9%, *n* = 39, 95% CI: 56.9–81.9%) followed by *An. pharoensis* (21.8%, *n* = 12; 95% CI: 12.2–35.4%). The proportion of *An. arabiensis* collected in the calf-baited MET was twofold higher than that in the human-baited MET (GLMM: OR = 2.36, *P* = 0.013). For *An. funestus * s.s., the proportion of blood-fed mosquitoes collected was inadequate in our view to detect a reliable difference between host types (GLMM: *P* = 0.614; Table 3). Moreover, the proportion of *An. coustani* (77%) caught in the calf-baited MET was significantly higher than that in the human-baited MET (GLMM: OR = 0.30, *P* = 0.001). The proportion of *An. arabiensis* caught in the METs was significantly higher in the cattle-baited traps in the early wet season and during the dry season (*χ*^2^_Mar_ = 25.8, *χ*^2^_Apr_ = 19.7, *χ*^2^_Jul_ = 72.5; *P* < 0.0001), while by the mid to late wet season there was no difference between the host-baited traps (Fig. 5).

## Discussion

Information on the seasonal change in the abundance and host preference of primary and secondary malaria vectors, and how these contribute to malaria transmission, is required to strengthen and improve integrated vector management strategies in the context of the current state of residual transmission [10, 20, 21, 49, 50]. In this study, primary [23, 51, 52] and secondary [36, 53] malaria vectors exhibited similar, yet distinct, abundance and feeding patterns within the wet season, extending across the transition between the wet and dry seasons. The proportion of mosquitoes that fed on humans suggests that both primary and secondary vectors may differentially contribute to the risk of malaria transmission during the wet season, whereas only the primary vectors appear to contribute to the risk of transmission during the dry season. These findings provide additional information on the ecology of the primary and secondary vectors within the region, which needs to be taken into account when planning future local control efforts.

The seasonal variation in the abundance of both primary and secondary vectors driven by the availability of rainfall reflects what has been observed in other parts of the Kilombero valley, throughout Tanzania and in other regions of SSA [49, 53–60]. Of the primary vectors, *An. arabiensis*, the only member of the *An. gambiae* s.l. complex found in the Kilombero valley [51, 61], was consistently more abundant than *An. funestus* s.s. across the wet and dry seasons, as previously reported in this valley [58, 61] and other regions of SSA [59, 62–64]. The seasonal pattern in abundance of the main secondary vector, *An. coustani,* reflected that of *An. arabiensis* and *An. funestus*, with the peak of abundance following the height of the wet season and extending into the dry season [56]. While the population of the less abundant secondary vectors increased much earlier in the wet season, and reached the peak of abundance at a similar time as the primary vectors and *An. coustani,* these mosquito populations did not persist into the dry season. The observed change in vector abundance across seasons is likely explained by the variation in access to, and choice of, breeding habitat, and to a lesser extent the ability of the vector species to make use of the available blood meals. During the wet season, the overall numbers of anophelines collected outdoors doubled compared to those collected indoors, which is likely a result of the contribution of the secondary vector populations, which feed outside, predominantly on bovine blood.

Both the primary vectors, along with *An. coustani*, exhibit higher flexibility in their choice of breeding habitats, showing a tolerance to small stagnant pools of water [65–70], whereas the breeding sites of the other secondary vectors are closely linked to rainfall, large pools and flood plains [71, 72]. The rise in the secondary vector populations for *An. pharoensis and An. squamosus* in the early wet season (March and April) suggests that these species tolerate the heavy rain associated with the flushing off of, and the mechanical damage to, mosquito eggs and larvae. The sharp decline in the secondary vectors *An. pharoensis* and *An. squamosus* in July corresponded with the drying out of the valley, leading to mass decrease in large pools as breeding sites, while small, stagnant pools were maintained into the dry season as breeding sites for *An. coustani* and the primary vectors. The ability of the primary vectors to make use of smaller, human-made and/or stagnant bodies of water as breeding sites enables them to maintain their population numbers into the dry season, thereby suggesting that this is an important factor regulating malaria transmission during this period [65, 69, 70, 73–75].

Host blood meal prevalence varied across the seasons in the primary vectors, which was most evident in *An. arabiensis* and further supported by direct assessment of host preference. While a seasonal variation in the host blood meals in the secondary vectors was indicated, further analysIs is needed for confirmation due to the low abundance of these vectors. The overall feeding patterns observed in this study are consistent with those previously described for the primary and secondary vector species in this study, in regions with similar host availability [76–78]. Blood meal analysis, as a proxy for host preference, is confounded by host availability [27, 36]; therefore, we used a direct measure of host preference, the METs, to provide supporting evidence of the seasonal shift in host choice in *An. arabiensis* [79]. These data showed a shift from a preference for cattle in the early wet season to opportunistic feeding late in the wet season and then a return to the preference for cattle in the transition to the dry season. A plausible explanation for the change in host preference may be related to the need to maximize the lifetime reproductive success [80] as the habitat conditions for *An. arabiensis* worsen in the dry season [73, 75]. Alternatively, this seasonal shift in host preference cannot be specifically stated, and it may simply reflect an effect of experience of a previous blood meal by the malaria vectors [81]. The ongoing discussion of host preference in *An. arabiensis* may be obscured by this apparent seasonality, leading some studies to conclude that this primary vector is zoophilic, while others argue for it being opportunistic [36, 82]. Further studies are required to elucidate how this apparent change in host preference across seasons affects malaria transmission.

Households with higher numbers of cattle and sheep had higher anopheline activity and a higher number of human-fed malaria vectors, suggesting that the abundance of non-human hosts, particularly cattle, in the study area may increase human-feeding rate, as previously reported [83–85]. In situations in which livestock are kept close to humans, the presence of animals may increase the risk of human-biting simply through the cattle attracting mosquitoes to the general proximity [85–87]. In contrast, the presence of livestock at households has also been shown to reduce human-feeding rates [77, 88, 89]. These contradictory observations may be attributed to variations in host preference and abundance of the dominant vector species, as well as in the distance of livestock from households [90]. This result emphasizes the need for further area-wide studies that incorporate direct measures of host preference, human-biting rates and host availability prior to implementing zooprophylaxis as a control strategy [91, 92].

Of the other non-human hosts available, dogs and goats were represented more often in the blood meals of *An. arabiensis* and several secondary vectors towards the end of the wet season and into the dry season. *Anopheles arabiensis* and the secondary vectors appeared to feed opportunistically throughout their activity periods based on blood meal analysis. The opportunistic behavior for the secondary vectors reflects what was previously observed in other regions of Tanzania [18], but contradicts findings from Zambia [93]. The determination of the degree of host-driven versus opportunistic behavior within populations and species may be easily misidentified when using blood meal analysis and host census data [94], and may be exacerbated in mosquito species which are in low abundance. In contrast to the seemingly opportunistic behaviors demonstrated by the other anophelines in this study, *An. funestus* demonstrated a potential switch in host choice from cattle to human [76, 95, 96], culminating at the peak of the wet season, thereby emphasizing the increasing importance of this species in malaria transmission in the region [10, 97]. During the dry season, there was increased availability of human blood meal due to a reduced use of bed nets compared to the wet season.

All malaria vectors collected in this study were negative for malaria parasites, emphasizing the decreasing malaria transmission in the region, most notably from 14% in 2011 to 11% in 2017 [30, 31]. Furthermore, other recent studies have indicated the lack of sporozoite-positive malaria vectors in the area [29]. This decline in malaria prevalence is likely due to urbanization, improved house construction and use of LLINs in conjunction with the ongoing livestock-keeping lifestyle in the study area [23, 29, 88, 98, 99]. While the circumsporozoite protein ELISA used in these studies may underestimate the prevalence of malaria parasites, the mosquitoes identified are assuredly infectious, as compared to the more sensitive PCR approaches, which can contain the sporozoites from anywhere in the body [100].

## Conclusions

This study provides updated seasonal information on the abundance, blood meal sources and host preference of the primary and secondary vectors in the Kilombero valley. *Anopheles arabiensis* and *An. coustani* were identified as the most abundant primary and secondary vectors, respectively. *Anopheles arabiensis* clearly demonstrated seasonality in host preference, while blood meal analysis only indicated this for *An. funestus* s.s. and the secondary vectors. While no sporozoite positive vectors were identified, the demonstrated seasonal changes in human biting are likely to contribute to low prevalence of malaria in the area. Further studies are required to assess the role of seasonal shifts in host preference in malaria transmission, and to increase our understanding of the ecology of *An. funestus* species complex.

## Data Availability

The dataset used and/or analyzed, as well as the materials collected, during the current study are available from the corresponding author on reasonable request.

## Abbreviations

CDC: US Centers for Disease Control and Prevention
GLMM: Generalized linear mixed model
IRS: Indoor residual spraying
LLIN: Long-lasting insecticidal nets
MET: Mosquito electrocuting trap
SSA: Sub-Saharan Africa

## References

[1] Bhatt S, Weiss DJ, Cameron E, Bisanzio D, Mappin B, Dalrymple U (2015). The effect of malaria control on *Plasmodium falciparum* in Africa between 2000 and 2015. Nature.

[2] WHO. World malaria report 2020. 20 years of global progress and challenges. 2020. https://www.who.int/publications/i/item/9789240015791. Accessed 23 Sept 2021.

[3] Bhattarai A, Ali AS, Kachur SP, Mårtensson A, Abbas AK, Khatib R (2007). Impact of artemisinin-based combination therapy and insecticide-treated nets on malaria burden in Zanzibar. PLoS Med.

[4] Kim D, Fedak K, Kramer R (2012). Reduction of malaria prevalence by indoor residual spraying: a meta-regression analysis. Am J Trop Med Hyg.

[5] Protopopoff N, Wright A, West PA, Tigererwa R, Mosha FW, Kisinza W (2015). Combination of insecticide treated nets and indoor residual spraying in northern Tanzania provides additional reduction in vector population density and malaria transmission rates compared to insecticide treated nets alone: a randomised control trial. PLoS ONE.

[6] Protopopoff N, Mosha JF, Lukole E, Charlwood JD, Wright A, Mwalimu CD (2018). Effectiveness of a long-lasting piperonyl butoxide-treated insecticidal net and indoor residual spray interventions, separately and together, against malaria transmitted by pyrethroid-resistant mosquitoes: a cluster, randomised controlled, two-by-two factorial design trial. Lancet.

[7] Killeen GF, Chitnis N (2014). Potential causes and consequences of behavioural resilience and resistance in malaria vector populations: a mathematical modelling analysis. Malar J.

[8] Mzilahowa T, Chiumia M, Mbewe RB, Uzalili VT, Luka-Banda M, Kutengule A (2016). Increasing insecticide resistance in *Anopheles funestus* and *Anopheles arabiensis* in Malawi, 2011–2015. Malar J.

[9] Riveron JM, Tchouakui M, Mugenzi L, Menze BD, Chiang MC, Wondji CS, Manguin S, Dev V (2018). Insecticide resistance in malaria vectors: an update at a global scale. Towards malaria elimination-a leap forward.

[10] Govella NJ, Ferguson H (2012). Why use of interventions targeting outdoor biting mosquitoes will be necessary to achieve malaria elimination. Front Physiol.

[11] Sherrard-Smith E, Skarp JE, Beale AD, Fornadel C, Norris LC, Moore SJ (2019). Mosquito feeding behavior and how it influences residual malaria transmission across Africa. Proc Natl Acad Sci USA.

[12] Sinka ME, Bangs MJ, Manguin S, Rubio-Palis Y, Chareonviriyaphap T, Coetzee M (2012). A global map of dominant malaria vectors. Parasit Vectors.

[13] Adja AM, N’goran EK, Koudou BG, Dia I, Kengne P, Fontenille D (2011). Contribution of *Anopheles funestus*, *An. gambiae* and *An. nili* (Diptera: Culicidae) to the perennial malaria transmission in the southern and western forest areas of Côte d’Ivoire. Ann Trop Med Parasitol.

[14] Bayoh MN, Mathias DK, Odiere MR, Mutuku FM, Kamau L, Gimnig JE (2010). *Anopheles gambiae*: historical population decline associated with regional distribution of insecticide-treated bed nets in western Nyanza Province, Kenya. Malar J.

[15] Derua YA, Alifrangis M, Hosea KM, Meyrowitsch DW, Magesa SM, Pedersen EM (2012). Change in composition of the *Anopheles gambiae* complex and its possible implications for the transmission of malaria and lymphatic filariasis in north-eastern Tanzania. Malar J.

[16] Mwangangi JM, Mbogo CM, Orindi BO, Muturi EJ, Midega JT, Nzovu J (2013). Shifts in malaria vector species composition and transmission dynamics along the Kenyan coast over the past 20 years. Malar J.

[17] Cooke MK, Kahindi SC, Oriango RM, Owaga C, Ayoma E, Mabuka D (2015). ‘A bite before bed’: exposure to malaria vectors outside the times of net use in the highlands of western Kenya. Malar J.

[18] Afrane YA, Bonizzoni M, Yan G, Rodriguez-Morales AJ (2016). Secondary malaria vectors of sub-Saharan Africa: threat to malaria elimination on the continent?. Current topics in malaria.

[19] Ekoko WE, Awono-Ambene P, Bigoga J, Mandeng S, Piameu M, Nvondo N (2019). Patterns of anopheline feeding/resting behaviour and *Plasmodium* infections in North Cameroon, 2011–2014: implications for malaria control. Parasit Vectors.

[20] Epopa PS, Collins CM, North A, Millogo AA, Benedict MQ, Tripet F (2019). Seasonal malaria vector and transmission dynamics in western Burkina Faso. Malar J.

[21] Antonio-Nkondjio C, Kerah CH, Simard F, Awono-Ambene P, Chouaibou M, Tchuinkam T (2006). Complexity of the malaria vectorial system in Cameroon: contribution of secondary vectors to malaria transmission. J Med Entomol.

[22] Mustapha AM, Musembi S, Nyamache AK, Machani MG, Kosgei J, Wamuyu L (2021). Secondary malaria vectors in western Kenya include novel species with unexpectedly high densities and parasite infection rates. Parasit Vectors.

[23] Lwetoijera DW, Harris C, Kiware SS, Dongus S, Devine GJ, McCall PJ (2014). Increasing role of *Anopheles funestus* and *Anopheles arabiensis* in malaria transmission in the Kilombero Valley, Tanzania. Malar J.

[24] Kaindoa EW, Ngowo HS, Limwagu AJ, Tchouakui M, Hape E, Abbasi S (2019). Swarms of the malaria vector *Anopheles funestus* in Tanzania. Malar J.

[25] Matowo NS, Martin J, Kulkarni MA, Mosha JF, Lukole E, Isaya G (2021). An increasing role of pyrethroid-resistant *Anopheles funestus* in malaria transmission in the Lake Zone, Tanzania. Sci Rep.

[26] Qiu YT, van Loon JJ, Takken W, Knols BGJ (2010). Olfactory physiology of blood-feeding vector mosquitoes. Olfaction in vector-host interactions.

[27] Mlacha YP, Chaki PP, Muhili A, Massue DJ, Tanner M, Majambere S (2020). Reduced human-biting preferences of the African malaria vectors *Anopheles arabiensis* and *Anopheles gambiae* in an urban context: controlled, competitive host-preference experiments in Tanzania. Malar J.

[28] Pinda PG, Eichenberger C, Ngowo HS, Msaky DS, Abbasi S (2020). Comparative assessment of insecticide resistance phenotypes in two major malaria vectors, *Anopheles funestus* and *Anopheles arabiensis* in south-eastern Tanzania. Malar J.

[29] Finda MF, Limwagu AJ, Ngowo HS, Matowo NS, Swai JK, Kaindoa E (2018). Dramatic decreases of malaria transmission intensities in Ifakara, south-eastern Tanzania since early 2000s. Malar J.

[30] Harchut K, Standley C, Dobson A, Klaassen B, Rambaud-althaus C (2013). Over-diagnosis of malaria by microscopy in the Kilombero Valley, Southern Tanzania : an evaluation of the utility and cost-effectiveness of rapid diagnostic tests. Malar J.

[31] Thawer SG, Chacky F, Runge M, Reaves E, Mandike R, Lazaro S, et al. Sub-national stratification of malaria risk in mainland Tanzania: a simplified assembly of survey and routine data. Malar J. 2020;19:177. 10.1186/s12936-020-03250-4 PMC7206674 32384923

[32] Sriwichai P, Karl S, Samung Y, Sumruayphol S, Kiattibutr K, Payakkapol A (2015). Evaluation of CDC light traps for mosquito surveillance in a malaria endemic area on the Thai-Myanmar border. Parasit Vectors.

[33] Maia MF, Robinson A, John A, Mgando J, Simfukwe E, Moore SJ (2011). Comparison of the CDC Backpack aspirator and the Prokopack aspirator for sampling indoor-and outdoor-resting mosquitoes in southern Tanzania. Parasit Vectors.

[34] Kweka EJ, Mwang'onde BJ, Kimaro E, Msangi S, Massenga CP, Mahande AM (2009). A resting box for outdoor sampling of adult *Anopheles arabiensis* in rice irrigation schemes of lower Moshi, northern Tanzania. Malar J.

[35] Kweka EJ, Owino EA, Mwang'onde BJ, Mahande AM, Nyindo M, Mosha F (2011). The role of cow urine in the oviposition site preference of culicine and *Anopheles* mosquitoes. Parasit Vectors.

[36] Meza FC, Kreppel KS, Maliti DF, Mlwale AT, Mirzai N, Killeen GF (2019). Mosquito electrocuting traps for directly measuring biting rates and host-preferences of Anopheles *arabiensis* and *Anopheles funestus* outdoors. Malar J.

[37] Gillies M, Meillon D (1968). The Anophelinae of Africa south of the Sahara (Ethiopian Zoogeographical Region).

[38] Gillies MT, Coetzee M (1987). A supplement to the Anophelinae of Africa South of the Sahara.

[39] Scott JA, Brogdon WG, Collins FH (1993). Identification of single specimens of the *Anopheles gambiae* complex by the polymerase chain reaction. Am J Trop Med Hyg.

[40] Cornel AJ, Porter CH, Collins FH (1996). Polymerase chain reaction species diagnostic assay for *Anopheles quadrimaculatus* cryptic species (Diptera: Culicidae) based on ribosomal DNA ITS2 sequences. J Med Entomol.

[41] Koekemoer LL, Kamau L, Hunt RH, Coetzee M (2002). A cocktail polymerase chain reaction assay to identify members of the *Anopheles funestus* (Diptera: Culicidae) group. Am J Trop Med Hyg.

[42] Cohuet A, Simard F, Toto JC, Kengne P, Coetzee MA, Fontenille D (2003). Species identification within the *Anopheles funestus* group of malaria vectors in Cameroon and evidence for a new species. Am J Trop Med Hyg.

[43] Ponlawat A, Harrington LC (2005). Blood feeding patterns of *Aedes aegypti* and *Aedes albopictus* in Thailand. J Med Entomol.

[44] Lardeux F, Loayza P, Bouchité B, Chavez T (2007). Host choice and human blood index of *Anopheles pseudopunctipennis* in a village of the Andean valleys of Bolivia. Malar J.

[45] Wirtz RA, Zavala F, Charoenvit Y, Campbell GH, Burkot TR, Schneider I (1987). Comparative testing of monoclonal antibodies against *Plasmodium falciparum* sporozoites for ELISA development. Bull World Health Organ.

[46] Durnez L, Van Bortel W, Denis L, Roelants P, Veracx A, Trung HD (2011). False positive circumsporozoite protein ELISA: A challenge for the estimation of the entomological inoculation rate of malaria and for vector incrimination. Malar J.

[47] R Core Team. R: A language and environment for statistical computing. Vienna: R Foundation for Statistical Computing; 2016. https://www.R-project.org/.

[48] Brooks ME, Kristensen K, Van Benthem KJ, Magnusson A, Berg CW, Nielsen A (2017). glmmTMB balances speed and flexibility among packages for zero-inflated generalized linear mixed modeling. R J.

[49] Lemrabott MAO, Salem MSOA, Brahim KO, Brengues C, Rossignol M, Bogreau H (2018). Seasonal abundance, blood meal sources and insecticide susceptibility in major anopheline malaria vectors from southern Mauritania. Parasit Vectors.

[50] Sanei-Dehkordi A, Soleimani-Ahmadi M, Jaberhashemi SA, Zare M (2019). Species composition, seasonal abundance and distribution of potential anopheline vectors in a malaria endemic area of Iran: field assessment for malaria elimination. Malar J.

[51] Kaindoa EW, Mkandawile G, Ligamba G, Kelly-Hope LA, Okumu FO (2016). Correlations between household occupancy and malaria vector biting risk in rural Tanzanian villages: implications for high-resolution spatial targeting of control interventions. Malar J.

[52] Russell TL, Govella NJ, Azizi S, Drakeley CJ, Kachur SP, Killeen GF (2011). Increased proportions of outdoor feeding among residual malaria vector populations following increased use of insecticide–treated nets in rural Tanzania. Malar J.

[53] Kaindoa EW, Finda M, Kiplagat J, Mkandawile G, Nyoni A, Coetzee M (2018). Housing gaps, mosquitoes and public viewpoints: a mixed methods assessment of relationships between house characteristics, malaria vector biting risk and community perspectives in rural Tanzania. Malar J.

[54] Ayala D, Costantini C, Ose K, Kamdem GC, Antonio-Nkondjio C, Agbor JP (2009). Habitat suitability and ecological niche profile of major malaria vectors in Cameroon. Malar J.

[55] Kigadye ES, Nkwengulila G, Magesa SM, Abdulla S (2010). Diversity, spatial and temporal abundance of *Anopheles gambiae* complex in the Rufiji River basin, south-eastern Tanzania. Tanzan J Health Res.

[56] Mwanziva CE, Kitau J, Tungu PK, Mweya CN, Mkali H, Ndege CM (2011). Transmission intensity and malaria vector population structure in Magugu, Babati district in northern Tanzania. Tanzan J Health Res.

[57] Taye B, Lelisa K, Emana D, Asale A, Yewhalaw D (2016). Seasonal dynamics, longevity, and biting activity of anopheline mosquitoes in southwestern Ethiopia. J Insect Sci.

[58] Kaindoa EW, Matowo NS, Ngowo HS, Mkandawile G, Mmbando A, Finda M (2017). Interventions that effectively target *Anopheles funestus* mosquitoes could significantly improve control of persistent malaria transmission in south–eastern Tanzania. PLoS ONE.

[59] Amaechi EC, Ukpai OM, Ohaeri CC, Ejike UB, Irole-Eze OP, Egwu O (2018). Distribution and seasonal abundance of Anopheline mosquitoes and their association with rainfall around irrigation and non-irrigation areas in Nigeria. Cuadernos de Investigación UNED Res J.

[60] Demissew A, Hawaria D, Kibret S, Animut A, Tsegaye A, Lee MC (2020). Impact of sugarcane irrigation on malaria vector *Anopheles* mosquito fauna, abundance and seasonality in Arjo-Didessa. Ethiopia Malar J.

[61] Ngowo HS, Kaindoa EW, Matthiopoulos J, Ferguson HM, Okumu FO (2017). Variations in household microclimate affect outdoor-biting behaviour of malaria vectors. Wellcome Open Res.

[62] Minakawa N, Sonye G, Mogi M, Githeko A, Yan G (2002). The effects of climatic factors on the distribution and abundance of malaria vectors in Kenya. J Med Entomol.

[63] McCann RS, Ochomo E, Bayoh MN, Vulule JM, Hamel MJ, Gimnig JE (2014). Reemergence of *Anopheles funestus* as a vector of *Plasmodium falciparum* in western Kenya after long-term implementation of insecticide-treated bed nets. Am J Trop Med Hyg.

[64] Dear NF, Kadangwe C, Mzilahowa T, Bauleni A, Mathanga DP, Duster C (2018). Household-level and surrounding peri-domestic environmental characteristics associated with malaria vectors *Anopheles arabiensis* and *Anopheles funestus* along an urban–rural continuum in Blantyre, Malawi. Malar J.

[65] Koenraadt CJ, Githeko AK, Takken W (2004). The effects of rainfall and evapotranspiration on the temporal dynamics of *Anopheles gambiae ss* and *Anopheles arabiensis* in a Kenyan village. Acta Trop.

[66] Sattler MA, Mtasiwa D, Kiama M, Premji Z, Tanner M, Killeen GF (2005). Habitat characterization and spatial distribution of *Anopheles sp*. mosquito larvae in Dar es Salaam (Tanzania) during an extended dry period. Malar J.

[67] Fillinger U, Sombroek H, Majambere S, Van Loon E, Takken W, Lindsay SW (2009). Identifying the most productive breeding sites for malaria mosquitoes in the Gambia. Malar J.

[68] Gouagna LC, Dehecq JS, Girod R, Boyer S, Lempérière G, Fontenille D (2011). Spatial and temporal distribution patterns of *Anopheles arabiensis* breeding sites in La Reunion Island—multi-year trend analysis of historical records from 1996–2009. Parasit Vectors.

[69] Kenea O, Balkew M, Gebre-Michael T (2011). Environmental factors associated with larval habitats of anopheline mosquitoes (Diptera: Culicidae) in irrigation and major drainage areas in the middle course of the Rift Valley, Central Ethiopia. J Vector Borne Dis.

[70] Nambunga IH, Ngowo HS, Mapua SA, Hape EE, Msugupakulya BJ, Msaky DS (2020). Aquatic habitats of the malaria vector *Anopheles funestus* in rural south-eastern Tanzania. Malar J.

[71] Machault V, Gadiaga L, Vignolles C, Jarjaval F, Bouzid S, Sokhna C (2009). Highly focused anopheline breeding sites and malaria transmission in Dakar. Malar J.

[72] Getachew D, Balkew M, Tekie H (2020). *Anopheles* larval species composition and characterization of breeding habitats in two localities in the Ghibe River Basin, southwestern Ethiopia. Malar J.

[73] Imbahale SS, Paaijmans KP, Mukabana WR, Van Lammeren R, Githeko AK, Takken W (2011). A longitudinal study on *Anopheles* mosquito larval abundance in distinct geographical and environmental settings in western Kenya. Malar J.

[74] Mathania MM, Munisi DZ, Silayo RS (2020). Spatial and temporal distribution of *Anopheles* mosquito's larvae and its determinants in two urban sites in Tanzania with different malaria transmission levels. Parasite Epidemiol Control.

[75] Animut A, Negash Y (2018). Dry season occurrence of *Anopheles* mosquitoes and implications in Jabi Tehnan District, west Gojjam Zone. Ethiopia Malar J.

[76] Githeko AK, Service MW, Mbogo CM, Atieli FK, Juma FO. Origin of blood meals in indoor and outdoor resting malaria vectors in western Kenya. Acta Trop. 1994;58:307-16 10.1016/0001-706x(94)90024-8 7709869

[77] Mahande A, Mosha F, Mahande J, Kweka E (2007). Feeding and resting behaviour of malaria vector, *Anopheles arabiensis* with reference to zooprophylaxis. Malar J.

[78] Jaleta KT, Hill SR, Birgersson G, Tekie H, Ignell R (2016). Chicken volatiles repel host-seeking malaria mosquitoes. Malar J.

[79] Kreppel KS, Viana M, Main BJ, Johnson PC, Govella NJ, Lee Y (2020). Emergence of behavioural avoidance strategies of malaria vectors in areas of high LLIN coverage in Tanzania. Sci Rep.

[80] Lyimo IN, Haydon DT, Russell TL, Mbina KF, Daraja AA, Mbehela EM (2013). The impact of host species and vector control measures on the fitness of African malaria vectors. Proc Biol Sci.

[81] Vantaux A, Lefèvre T, Dabiré KR, Cohuet A (2014). Individual experience affects host choice in malaria vector mosquitoes. Parasit Vectors.

[82] Perugini E, Guelbeogo WM, Calzetta M, Manzi S, Virgillito C, Caputo B (2020). Behavioural plasticity of *Anopheles coluzzii* and *Anopheles arabiensis* undermines LLIN community protective effect in a Sudanese-savannah village in Burkina Faso. Parasit Vectors.

[83] Seyoum A, Balcha F, Balkew M, Ali A, Gebre-Michael T (2002). Impact of cattle keeping on human biting rate of anopheline mosquitoes. East Afr Med J.

[84] Tirados I, Costantini C, Gibson G, Torr SJ (2006). Blood-feeding behaviour of the malarial mosquito *Anopheles arabiensis*: implications for vector control. Med Vet Entomol.

[85] Hasyim H, Dhimal M, Bauer J, Montag D, Groneberg DA, Kuch U (2018). Does livestock protect from malaria or facilitate malaria prevalence? A cross-sectional study in endemic rural areas of Indonesia. Malar J.

[86] Zeru MA, Shibru S, Massebo F (2020). Exploring the impact of cattle on human exposure to malaria mosquitoes in the Arba Minch area district of southwest Ethiopia. Parasit Vectors.

[87] Bouma M, Rowland M (1995). Failure of passive zooprophylaxis: cattle ownership in Pakistan is associated with a higher prevalence of malaria. Trans R Soc Trop Med Hyg.

[88] Mayagaya VS, Nkwengulila G, Lyimo IN, Kihonda J, Mtambala H, Ngonyani H (2015). The impact of livestock on the abundance, resting behaviour and sporozoite rate of malaria vectors in southern Tanzania. Malar J.

[89] Mburu MM, Zembere K, Mzilahowa T, Terlouw AD, Malenga T, van den Berg H (2021). Impact of cattle on the abundance of indoor and outdoor resting malaria vectors in southern Malawi. Malar J.

[90] Saul A (2003). Zooprophylaxis or zoopotentiation: the outcome of introducing mortality while searching. Malar J.

[91] Iwashita H, Dida GO, Sonye GO, Sunahara T, Futami K, Njenga S (2014). Push by a net, pull by a cow: can zooprophylaxis enhance the impact of insecticide treated bed nets on malaria control?. Parasit Vectors.

[92] Asale A, Duchateau L, Devleesschauwer B, Huisman G, Yewhalaw D (2017). Zooprophylaxis as a control strategy for malaria caused by the vector *Anopheles arabiensis* (Diptera: Culicidae): a systematic review. Infect Dis Poverty.

[93] Fornadel CM, Norris LC, Franco V, Norris DE (2011). Unexpected anthropophily in the potential secondary malaria vectors *Anopheles coustani s.l*. and *Anopheles squamosus* in Macha, Zambia. Vector-Borne Zoonotic Dis..

[94] Govella NJ, Maliti DF, Mlwale AT, Masallu JP, Mirzai N, Johnson PCD (2016). An improved mosquito electrocuting trap that safely reproduces epidemiologically relevant metrics of mosquito human-feeding behaviours as determined by human landing catch. Malar J.

[95] Mwangangi JM, Mbogo CM, Nzovu JG, Githure JI, Yan G, Beier JC (2003). Blood-meal analysis for anopheline mosquitoes sampled along the Kenyan coast. J Am Mosq Control Assoc.

[96] Degefa T, Yewhalaw D, Zhou G, Lee MC, Atieli H, Githeko AK (2017). Indoor and outdoor malaria vector surveillance in western Kenya: implications for better understanding of residual transmission. Malar J.

[97] WHO. World Health Organization. World malaria report 2008. 2008. https://apps.who.int/iris/handle/10665/43939 Accessed 29 Jul 2021

[98] Bulterys PL, Mharakurwa S, Thuma PE (2009). Cattle, other domestic animal ownership, and distance between dwelling structures are associated with reduced risk of recurrent *Plasmodium falciparum* infection in southern Zambia. Trop Med Int Heal.

[99] Drakeley C, Schellenberg D, Kihonda J, Sousa CA, Arez AP, Lopes D (2003). An estimation of the entomological inoculation rate for Ifakara: a semi-urban area in a region of intense malaria transmission in Tanzania. Trop Med Int Heal.

[100] Hendershot AL, Esayas E, Sutcliffe AC, Irish SR, Gadisa E, Tadesse FG (2021). A comparison of PCR and ELISA methods to detect different stages of *Plasmodium vivax* in *Anopheles arabiensis*. Parasit Vectors.

